# Neuroinflammatory Modulation of Extracellular Vesicle Biogenesis and Cargo Loading

**DOI:** 10.1007/s12017-022-08704-3

**Published:** 2022-02-18

**Authors:** Jereme G. Spiers, Natasha Vassileff, Andrew F. Hill

**Affiliations:** Department of Biochemistry and Genetics, La Trobe Institute for Molecular Science, La Trobe University, Bundoora, VIC 3083 Australia

**Keywords:** Extracellular vesicles, Neuroinflammation, Cell biology

## Abstract

Increasing evidence suggests neuroinflammation is a highly coordinated response involving multiple cell types and utilising several different forms of cellular communication. In addition to the well documented cytokine and chemokine messengers, extracellular vesicles (EVs) have emerged as key regulators of the inflammatory response. EVs act as vectors of intercellular communication, capable of travelling between different cells and tissues to deliver selectively packaged protein, miRNA, and lipids from the parent cell. During neuroinflammation, EVs transmit specific inflammatory mediators, particularly from microglia, to promote inflammatory resolution. This mini-review will highlight the novel neuroinflammatory mechanisms contributing to the biogenesis and selective packaging of EVs.

## Neuroinflammation

Neuroinflammation is a common feature of neurodegenerative conditions, such as Alzheimer’s and Parkinson’s diseases, manifesting early in disease pathogenesis and exacerbating progression by promoting neuronal death (Bourgognon et al., 2021). Additionally, neuroinflammation has been observed in neurological disorders including epilepsy, schizophrenia, and depression (Brites & Fernandes, 2015; Cukovic et al., 2021; Woo et al., 2020). Principally, this is driven by polarisation and proliferation of glial cells, of which both astrocytes and microglia have distinct activation profiles promoting pro-inflammatory phenotypes (Liddelow et al., 2017). In microglia, activation can be broadly characterised into M1 inflammatory and M2 anti-inflammatory polarisation states, with the overall microglial phenotype depending heavily on the context of the activating signal (Rőszer, 2015). More specifically, microglial activation exhibits a highly dynamic and often overlapping spectrum, with sub-categories of M2 activation arising from a diverse range of inflammatory mediators (Rőszer, 2015). These different activation states allow microglia to respond by inducing or resolving inflammation which can be either deleterious or beneficial to neurons (Rőszer, 2015). In astrocytes, neuroinflammation induces a reactive phenotype characterised by the production of pro-inflammatory cytokines and reactive oxygen species (ROS) which induce toxicity in neurons and oligodendrocytes (Li et al., 2019). Unlike aforementioned glial cells, oligodendrocytes do not adopt pro-inflammatory phenotypes, instead responding to neuroinflammation through hypomyelination and necrosis (Li et al., 2008; Pang et al., 2003). Cytokines and chemokines are key signalling molecules involved in inflammation, coordinating glial activation and, under pro-inflammatory conditions, promoting the phenotypic transformation into migratory ameboid phagocytic cells (Rőszer, 2015). As the resident macrophages of the CNS, microglia sensing a threat or injury activate pro-inflammatory signalling cascades including the Nuclear Factor Kappa Light Chain Enhancer of Activated B cells (NF-κB) pathway, resulting in production of cytokines including Tumour Necrosis Factor-α (TNF-α), Interleukin-6 (IL-6), and Interleukin-1β (IL-1β) (Wang et al., 2015). The release of these cytokines into the surrounding tissues creates a feed-forward loop, activating surrounding microglia via cognate TNF-α/ IL-1β receptors and promoting further inflammation (Glass et al., 2010).

The complement cascade is another arm of the innate immune system that regulates inflammation in neuroinflammatory conditions and in response to invading pathogens. Although the complement cascade is intrinsically linked with cytokine signalling, complement signalling can be activated independently, with the most potent downstream effectors of the complement cascade, complement component C3a and C5a, acting collectively as anaphylotoxins, chemokines, and cytokine precursors (Merle et al., 2015; Tang et al., 2012). In the brain, these complement components are largely expressed in glial cells, playing a crucial role promoting astrocyte reactivity and microglial polarisation in neuroinflammatory conditions (Clarke et al., 2018; Zhang et al., 2020). In addition to cytokine signalling, chemokine-driven cell–cell communication has emerged as a critical mediator of normal physiological and neuroinflammatory disease-related processes. Recent studies have identified neuron-microglia crosstalk via chemokines such as fractalkine enables neurons to directly modulate microglial activation (Dutta et al., 2020). Neurons constitutively express the fractalkine ligand (CX3CL1), which binds to microglial CX3CR1 receptors found exclusively in microglia, inhibiting microglial activation (Pawelec et al., 2020). Reductions in fractalkine signalling, observed in neuroinflammatory lipopolysaccharide (LPS) treatment and neurodegenerative disease models, result in potentiated microglial activation and pro-inflammatory signalling (Pawelec et al., 2020). Additionally, neuronal and glial cell types express toll-like receptors (TLRs), including TLR-4, which also leads to activation of the NF-κB pathway and plays a vital role in cell survival during infection (Mishra et al., 2006). However during neuroinflammation, overactivation of the TLR-4 pathway induces neuronal death which can further promote TLR-4 activation via damage-associated molecular pattern (DAMP) recognition to exacerbate this condition (Calvo-Rodríguez et al., 2017; Mishra et al., 2006).

**Fig. 1:**
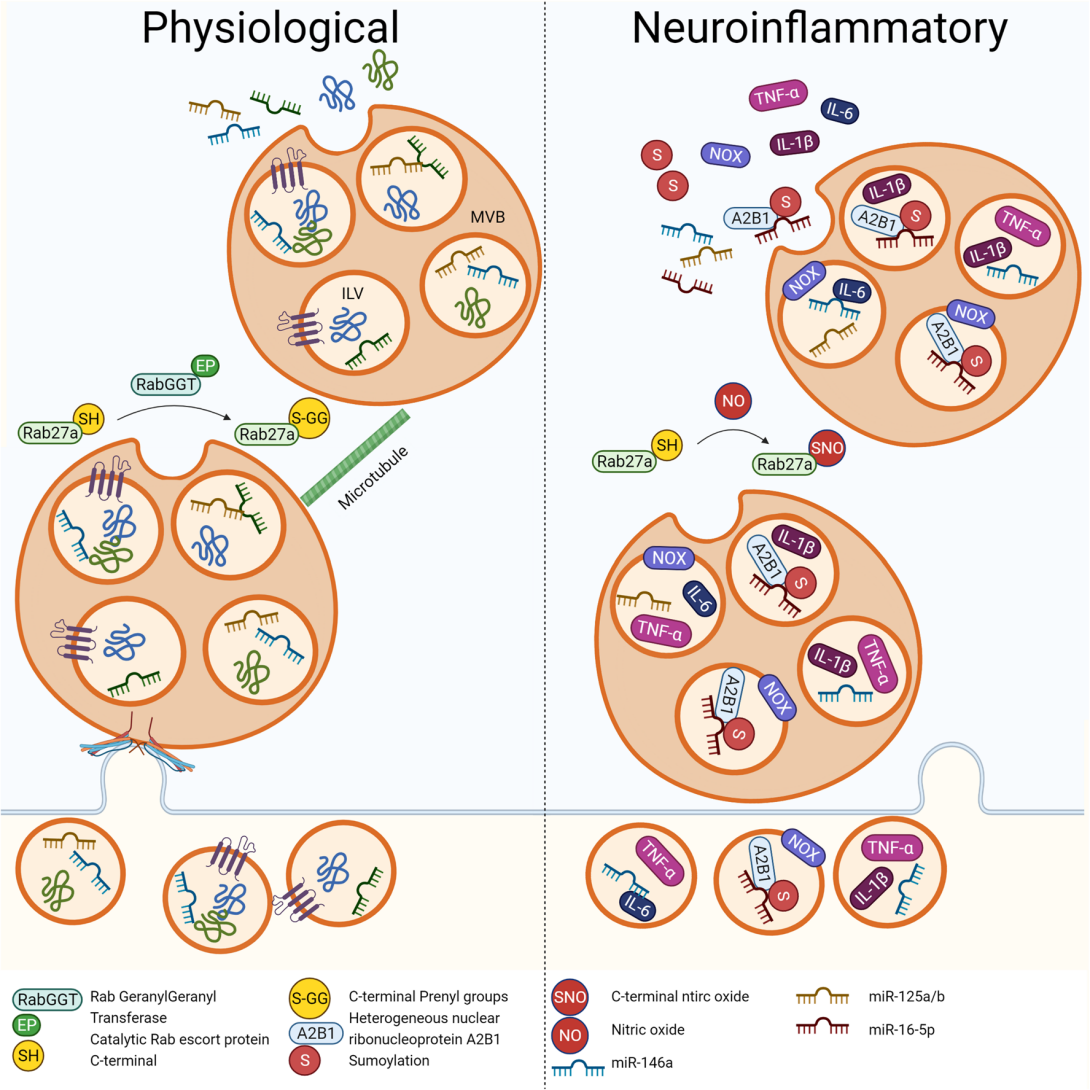
Neuroinflammatory modulation of EV biogenesis and loading. Under physiological conditions, cargo including protein and RNA is transferred to the limiting membrane of the multivesicular body (MVB) resulting in the formation of intraluminal vesicles (ILVs). The MVB is then guided via microtubules to the plasma membrane resulting in the release of these ILVs as exosomes. This process is assisted by Rab27a, following prenylation of it’s C-terminus by Rab GeranylGeranyl Transferase and its catalytic Rab escort protein. However under neuroinflammary conditions, increased nitric oxide (NO) may outcompete prenylation of the C-terminal residues on Rab27a through S-nitrosylation, impeding its ability to assist in exosome biogenesis via this pathway. Additionally, inflammation induces sumoylation of ribonucleoprotein A2B1, which is then packaged into the ILVs along with it’s immunomodulatory target miRNA allowing for post-transcriptional regulation of inflammation. Finally, pro-inflammatory molecules including Tumour Necrosis Factor-α (TNF-α), Interleukin-6 (IL-6), Interleukin-1β (IL-1β), and NADPH-Oxidase (NOX), are preferentially packaged into these exosomes, which contributes to neuroinflammatory inter-cellular communication. Figure created using Bio Render

Another critical pro-inflammatory pathway upregulated during microglial activation and increased NF-κB signalling is the suite of oxidative and nitrosative stress machinery capable of producing ROS and reactive nitrogen species (RNS) (Chen et al., 2015). Whilst each cell has a basal metabolic production of ROS, particularly superoxide, via normal mitochondrial oxidative phosphorylation, microglia also upregulate professional ROS/RNS producing enzymes, including NADPH-Oxidase (NOX) and inducible nitric oxide synthase (iNOS) (Chen et al., 2015). The NOX family of enzymes is one of the primary cellular sources of oxidants, producing superoxide radicals, and thereby the superoxide downstream enzymatic degradation products such as hydrogen peroxide and hydroxyl radical, following the highly regulated assembly of the NOX holoenzyme (Petersen et al., 2021). The iNOS enzyme can produce large quantities of nitric oxide (NO) in the absence of co-factors. This NO can traverse membranes in a diffusion-limited manner to exert concentration-dependent effects in neighbouring cells. Typically, acute production of these reactive molecules would be used to attack invading pathogens and promote the resolution of inflammation (Fang & Vázquez-Torres, 2019). However, the long-term production of ROS/RNS damages neurons due to their relative paucity of antioxidant protection in conjunction with their high oxygen consumption. High levels of NO also increase the likelihood of superoxide radical interaction and spontaneous formation of peroxynitrite, a potent neurotoxin. Moreover, high NO concentrations can attack cysteine and tyrosine amino acid side chains to form nitrosylated and nitrotyrosinated protein post-translational modifications (PTMs), resulting in altered function and localisation of these proteins (Bradley & Steinert, 2016). Inflammatory and oxidative mediators can also undergo both post-transcriptional regulation by microRNA (miRNA), and post-translational regulation via a suite of enzymatic modifications (Scalavino et al., 2020). Together, these mechanisms enable tight temporal and sub-cellular distribution control over neuroinflammatory processes to constrain neuronal damage and promote the resolution of inflammation. However, neuroinflammatory processes such as those observed in neurodegenerative conditions can also be transmitted over long distances, including to different regions of the brain or different tissues entirely, by hijacking other mechanisms of inter-cellular communication (Fig. 1).

## Extracellular Vesicles

Extracellular vesicles (EVs) are membraneous vesicles released by all cell types and encompass small EVs (sEVs) or exosomes, medium EVs (mEVs) or microvesicles, and large EVs (lEVs) or apoptotic bodies, with the former two being well documented in facilitating cell–cell communication. Despite overlaps in their functions, exosomes and microvesicles have key characteristics attributed to their unique biogenesis processes (Vassileff et al., 2020). Exosomes begin their biogenesis when early endosomes, containing endocytosed material, mature into late endosomes a process assisted by nSMase2 (Stoorvogel et al., 1991). During this process, cargo including protein, RNA, and lipids are sorted to the endosome's limiting membrane, causing it to invaginate and form intraluminal vesicles (ILVs) (Valadi et al., 2007). The presence of these vesicles dictates the maturation of the endosome into a multivesicular body (MVB) (Vassileff et al., 2020). The sorting and encapsulation of cargo are controlled by endosomal sorting complexes required for transport (ESCRT) proteins or ESCRT-independent mechanisms, lipids, and tetraspanins (Andreu & Yáñez-Mó, 2014). The MVB then fuses with either a lysosome, resulting in degradation of the ILVs, or with the plasma membrane, resulting in the release of ILVs as exosomes, a process controlled by the Rab GTPases: Rab27a and Rab27b and assisted by SNAP-23 in neuronal cells (Vassileff et al., 2020). Alternatively, microvesicle biogenesis begins when a stimulus initiates an increase in intracellular calcium (Ca^2+^) (Vassileff et al., 2020). The Ca^2+^ activates cytoskeletal remodelling proteins and induces plasma membrane phospholipids to become asymmetrically distributed, allowing for the redistribution of select biomolecules, a process involving ARF6 and specific ESCRT proteins (Greening et al., 2017). Lipid translocases maintain the phospholipid asymmetry until the increasing Ca^2+^ levels recruit contractile and scission proteins, which cleave cytoskeletal proteins, further destabilising the attachment of the plasma membrane to the cytoskeleton (Vassileff et al., 2020). This leads to outward budding of the plasma membrane and the subsequent shedding of microvesicles (Greening et al., 2017; Vassileff et al., 2020). Both exosomes and microvesicles can traverse long distances to interact with recipient cells, ultimately delivering their contents following endocytosis (Vassileff et al., 2020).

## Inflammation and Extracellular Vesicles

Under conditions promoting cellular stress, microglia have been documented to upregulate exosomal loading of miR-146a and miR-125b, known to modulate the NF-κB pathway and regulate microglial activation (Vaz et al., 2019). Furthermore, pro-inflammatory mediators, including IL-1β, IL-6, TNF-α, and caspase-1, have been detected in the cargo of EVs during inflammation (Kumar et al., 2017). Conversely, mEVs may play a more active role in inflammation, being found to initiate the inflammatory response by activating TLR-4 receptors on macrophages via the lipid component of their membranes (Kumar et al., 2017). In neurodegenerative diseases and neurological disorders, TLR-4 expression induces microglial cytokine and ROS production in addition to increasing EV loading of TLR-4 (Bhargava et al., 2019; Ibáñez et al., 2019). Similarly, mEVs have been found to carry complement proteins including C1q, C4, C3, and pentameric C-reactive protein (pCRP) on their surface alluding to their ability to also activate the classical complement pathway in recipient cells via their membranes (Biró et al., 2007; Guha et al., 2019). Furthermore, LPS activated monocytic mEVs incubated with CRP increased ICAM-1 and VCAM-1 expression in recipient cells, mediating recruitment of inflammatory leukocytes (Braig et al., 2017). Additionally, pro-inflammatory cytokines released during inflammation have been documented to stimulate the increase in Ca^2+^ responsible for initiating microvesicle biogenesis (Greening et al., 2017). Inflammation is also known to alter the loading and biogenesis of EVs. TNF-α, and IL-1β-induced activation of astrocytes can upregulate EV loading of miR-34a, miR-125a-5p, and miR-16-5p, which target Bcl-2 and the NT-3 growth factor receptor (Chaudhuri et al., 2018). These IL-1β-induced astrocytic EVs are preferentially taken up by recipient neurons, where they impair synaptic stability, reduce excitability, and increase neurotoxin vulnerability (Chaudhuri et al., 2018; Datta Chaudhuri et al., 2020; Mao et al., 2015; You et al., 2019). Furthermore, LPS-treated astrocytes and microglia release factors that delay oligodendrocytic maturation and invoke hypomyelination via calcium and cytokine dependent pathways including TNF-α. The transportation of these factors to oligodendrocytes has been suggested to involve EVs (Li et al., 2008; Pang et al., 2003; Peferoen et al., 2014; Sherwin & Fern, 2005). Therefore, EVs exhibit an interconnected relationship with inflammation that exceeds simply mediating the inflammatory response.

EVs may also be a novel mechanism promoting mobilisation and deployment of cell-type specific oxidative machinery during inflammation. Microglial NOX proteins are compartmentalised into sEVs following endosomal sorting, and this is suggested to form part of an intracellular secretory vesicle pool released following stimulation (Ejlerskov et al., 2012). These sEVs can be released following inflammatory signalling via TNF-α, allowing cells to communicate NOX oxidant producing capacity to neighbouring cells and tissues (Ejlerskov et al., 2012). Interestingly, these NOX sEVs also demonstrate oxidant production despite the lack of cytosolic phox-subunit cycling or sustainable NADPH substrate source required for maintaining enzyme activity (Petersen et al., 2021). These studies highlight that NOX and its corresponding cytosolic phox-subunit proteins undergo specific sorting into sEVs by an as yet undefined mechanism, presumably upregulated by pro-inflammatory signalling.

## Pro-inflammatory Modulation of Extracellular Vesicle Cargo

EV cargo loading offers a profound and relatively untapped area of therapeutic target discovery due to the critical role this process plays in neuroinflammatory and neurodegenerative disease pathogenesis. Despite this, relatively little is known about these mechanisms and how they are altered in neuroinflammation. Broadly, EV cargo loading and release can be modulated during microglial activation via inflammatory regulation of EV biosynthetic gene expression. The proteome of microglial EVs is also highly dependent on the activation state and stimulus context of the parental microglia, with EVs produced from activated microglia expressing high levels of TNF-α and promoting neuronal death (Chang et al., 2013).

Another emerging mechanism responsible for regulating EV cargo loading is the specific addition of PTMs to proteins targeted for inclusion into EVs and those involved in EV biogenesis. The aforementioned Rab27a, known to be involved in exosome biogenesis, is also a target of protein PTMs, most notable of which includes prenylation with either the farnesyl or geranylgeranyl pyrophosphate groups (Shinde & Maddika, 2018). Despite lacking the typical prenylating C-terminal CAAX motif, Rab proteins are targeted for prenylation by a specific Rab GeranylGeranyl Transferase that utilises a catalytic Rab escort protein to facilitate the transfer of two prenyl groups to the two C-terminal cysteine residues (Shinde & Maddika, 2018). Replacement of the Rab27a terminal dicysteine with a monocysteine resulted in mistargeting of the mutant protein and failed to restore wild type function in Rab27a^−/−^ cells, highlighting that the presence of two prenylating moieties is critically important for normal protein function (Shinde & Maddika, 2018). Under conditions of increased NO production, such as those observed in neuroinflammation, NO has been shown to compete with prenyl moieties via S-nitrosylation of terminal cysteine residues to regulate protein membrane localisation function, and ultimately vesicular release (Robinson et al., 2018). The presence of these terminal cysteine motifs across several members of the Ras superfamily, including Rab GTPases, suggests S-nitrosylation could broadly regulate vesicular trafficking and biogenesis during inflammation. There are also several protein PTMs involved in subcellular trafficking of cargo proteins into sEVs.

Interestingly, using combined stimulation with phorbol 12-myristate 13-acetate (PMA)/ionomycin, a protein kinase C activator able to induce pro-inflammatory cytokine production and a calcium ionophore, respectively, sumoylating protein PTMs were identified as a critical exosome cargo sorting mechanism in inflammation (Villarroya-Beltri et al., 2013). Pro-inflammatory PMA treatment induced sumoylation of heterogeneous nuclear ribonucleoprotein A2B1, a protein with high affinity for a specific motif of miRNA known to modulate inflammation and found to localise into exosomes (Villarroya-Beltri et al., 2013). This suggests EVs play an active role in recruiting and communicating signals capable of modulating inflammation at a post-transcriptional level.

In conclusion, EVs are emerging as critical players in the neuroinflammatory response, conveying key signalling molecules between different cell and tissue types. These are themselves regulated by neuroinflammatory mediators, highlighting the need for further study into the mechanism of EV biogenesis and cargo loading.
